# Effect of steroid eyedrops after trabeculectomy in glaucoma patients: a keratograph analysis

**DOI:** 10.5935/0004-2749.20210050

**Published:** 2021

**Authors:** Nikoly T. Fares, Renata C. Portela, Lilian F. Machado, Murilo Polizelli, Denise de Freitas, Augusto Paranhos Jr, Tiago S. Prata, Carolina P. B. Gracitelli

**Affiliations:** 1 Department of Ophthalmology and Visual Science, Glaucoma Service, Universidade Federal de São Paulo, São Paulo, Brazil; 2 Glaucoma Unit, Hospital Medicina dos Olhos, Osasco, SP, Brazil; 3 Department of Ophthalmology, Mayo Clinic, Jacksonville, FL, USA; 4 Department of Ophthalmology, Glaucoma Service, Hospital Oftalmológico de Sorocaba, Sorocaba, SP, Brazil; 5 Glaucoma Unit, Ver Mais Oftalmologia, Vinhedo, SP, Brazil

**Keywords:** Glaucoma, Ophthalmic solutions, Ocular surface di seases, Trabeculectomy, Glaucoma, Soluções oftálmicas, Doença de superfície ocular, Trabeculectomia

## Abstract

**Purpose:**

To investigate the use of preoperative steroid eyedrops in glaucoma patients undergoing trabeculectomy for ocular surface disease.

**Methods:**

A total of 31 eyes of 31 glaucoma patients were included. Only glaucoma patients who had been using at least three topical intraocular pressure-lowering medications for longer than 6 months were included. All patients were treated with loteprednol etabonate ophthalmic suspension 0.5% four times per day for 1 week before trabeculectomy. Data from baseline (day of surgery) and the follow-up visit (2 weeks after surgery) were included. All patients underwent a detailed ophthalmologic examination. Ocular surface disease was evaluated using the Ocular Surface Disease Index questionnaire and clinical measures, including tear breakup time, conjunctival hyperemia, and biomicroscopy to detect the presence or absence of keratitis. Ocular Surface Disease Index scores greater than 13 indicated a clinically relevant presence of ocular surface disease. In addition, all patients underwent keratograph analysis. The comparison of ocular surface disease before and after trabeculectomy was assessed using a paired test.

**Results:**

The mean age of the glaucoma patients was 69.90 ± 10.77 years. The average visual acuity was 0.40 ± 0.34 logMAR. The overall Ocular Surface Disease Index prevalence rate was 27.20 ± 17.56 units. Clinical assessment revealed no significant difference in bulbar redness, breakup time, or keratitis before and after surgery (p>0.05 for all comparisons). Keratograph analysis showed that the only two parameters that were significantly different before and after trabeculectomy ewere the bulbar redness by keratograph (BR-K) and the average noninvasive tear breakup time. Patients presented more conjunctival hyperemia and shorter noninvasive tear breakup time after trabeculectomy as compared with before surgery (p=0.013 and *p*=0.041, respectively).

**Conclusions:**

The present study not only confirms the high prevalence of clinical findings of ocular surface disease in glaucoma patients but also reveals new objective parameters measured by keratograph analysis. Apart from using loteprednol etabonate ophthalmic suspension 0.5% 1 week before the surgery, our sample presented a worsening of conjunctival hyperemia (bulbar redness by keratograph) and also a shorter noninvasive tear breakup time postoperatively.

## Abstract (pt)

**Objetivo:**

Investigar a ação de colírios esteróides na doença de superfície ocular em pacientes a serem submetidos a cirurgia de trabeculectomia.

**Métodos:**

Foram incluídos 31 olhos de 31 pacientes com glaucoma em uso de pelo menos 3 medicações tópicas antihipertensivas há mais de 6 meses. Todos os pacientes foram tratados com colírio de etabonato de loteprednol 0,5% (1 gota a cada 6 horas) durante os 7 dias precedentes à cirurgia de trabeculectomia. Além disso, foram submetidos a exame oftalmológico completo e responderam questionário validado que visa avaliar parâmetros subjetivos correlacionados a doenças da superfície ocular (Ocular Surface Disease Index). Os aspectos clínicos avaliados foram: tempo de ruptura lacrimal, coloração da córnea após colírio de fluoresceína (ceratite) e hiperemia conjuntival. Os pacientes foram, ainda, submetidos à análise da superfície ocular através de novo software tecnológico denominado “Keratographic”, tecnologia não invasiva que permite avaliar a doença da superfície ocular. A comparação da doença de superfície ocular antes e após a trabeculectomia foi avaliada estatisticamente através do teste pareado.

**Resultados:**

A média de idade dos participantes foi de 69,90 ± 10,77 anos. A AV média foi de 0,40 ± 0,34 logMAR. A taxa de prevalência global da Ocular Surface Disease Index foi de 27,20 ± 17,56 unidades. Em relação à avaliação clínica, não houve diferença significativa em relação hiperemia, ruptura lacrimal e ceratite antes e após a cirurgia (*p*>0,05 para todas as comparações). Em relação à análise com o “keratograph (menisco lacrimal, hiperemia, tempo de ruptura do filme lacrimal, meibografia para a pálpebra superior e inferior), os dois únicos parâmetros que diferiram significativamente antes e após a trabeculectomia, foram hiperemia e a média do tempo de rupture do filme lacrimal. Após a cirurgia de trabeculectomia, os pacientes apresentaram aumento da hiperemia conjuntival e diminuição do tempo de ruptura do filme lacrimal (p=0,013 e p=0,041, respectivamente).

**Conclusões:**

O presente estudo, não somente confirma a elevada prevalência da doença de superfície ocular em pacientes com glaucoma, como também demonstra que a mesma pode ser mensurada objetivamente através de parâmetros mensurados pelo Keratograph. Apesar de ter utilizado etabonato de loteprednol 0,5% uma semana antes da cirurgia, nossa amostra apresentou piora da hiperemia conjuntival e diminuição no tempo de ruptura do filme lacrimal no pós-operatório.

## INTRODUCTION

Glaucoma is a leading cause of irreversible blindness and visual impairment^(1)^. The disease is characterized by progressive optic nerve changes that may lead to loss of visual function and decrease in vision-related quality of life (QoL)^(1-4)^. Epidemiological studies estimate that approximately 60.5 million people worldwide have glaucoma, and it is predicted that the number will increase to 79.6 million by 2020, mostly because of the rapidly aging population^(3)^. Different parameters are associated with loss of QoL in these patients, including the use of eyedrop medications or the need for numerous surgical procedures in their eyes^(2,3,5)^.

The use of therapies that lower ocular pressure has already been reported to been associated with ocular surface disease (OSD)^(6,7)^. Some patients may have irritation, burning, ocular dryness, lacrimation, foreign-body sensation, red eye, or blurred vision. It is known that both the active principle of ocular hypotensive eye drops and the preservative used, usually benzalkonium chloride (BAK), can cause and/or aggravate changes in the ocular surface^(6,7)^. Careful observation is needed particularly for eyes that are treated with multiple eye drops, especially in older patients or those who have additional eye problems^(8)^. A previous reported that moderate or severe OSD affected 38% of patients who re ceived a single topical therapy, 54% of those who received two topical therapies, and 71% of those who received three or more topical therapies^(9)^. Previous studies have also investigated the relationship between OSD and surgical procedures in patients with glaucoma^(9,10)^. One of the most concerning effects of the subclinical inflammation caused by glaucoma medication is the failure of filtration surgery, which is frequently the last alternative in the treatment of glaucoma^(9,10)^. Baudouin et al. and Johnson et al. demonstrated that the duration and number of glaucoma medications used by the patient directly affect filtration surgery. In addition, several studies have demonstrated that the preoperative hypercellularity of chronic inflammatory cells (including fibroblasts, macrophages, and lymphocytes) of the trabecular meshwork is greatly reduced in patients who have undergone successful surgeries^(11-13)^. Therefore, there is solid ground to assume that the presence of the chronic inflammatory response associated with preservative toxicity, specifically BAK toxicity, may cause adverse surgical outcomes as a result of the fibrosis of the bleb, which in turn indicates a strong positive correlation between glaucoma medication and surgery failure^(9-12,13)^. Furthermore, corticosteroids have been reported to decrease symptoms of ocular irritation and corneal fluorescein staining in cases of OSD^(14)^. In a retrospective clinical series by Marsh and Pflugfelder, topical administration of a 1% solution of nonpreserved methylprednisolone, given three to four times daily for two weeks, to patients with Sjögren’s syndrome keratoconjunctivitis sicca (KCS) provided moderate to complete symptom relief in all patients^(15)^. This therapy was even effective for patients with severe KCS who demonstrated no improvement with maximum aqueous enhancement therapies^(15)^. In a prospective, randomized clinical trial by Sainz de la Maza et al., topical treatment of dry eye patients with nonpreserved methylprednisolone and punctual plugs significantly decreased the severity of ocular irritation symptoms and corneal fluorescein staining as compared with the group receiving punctual occlusion alone^(16)^.

Until now, elevated intraocular pressure (IOP) has been considered the major known risk factor for glaucoma progression^(5,7)^. IOP can be lowered using different methods, including topical medications, laser procedures, or incisional surgery^(13)^. Among incisional surgeries, trabeculectomy is the most frequently used, and it is effective for decreasing IOP^(9,10)^. However, the surgery can lead to some side effects, including OSD^(9,10)^. Thus, the purpose of this study was to investigate the use of preoperative steroid eyedrops for OSD in glaucoma patients undergoing trabeculectomy using subjective (e.g., Ocular Surface Disease Index [OSDI]) and objective (e.g., keratograph and clinical analysis) parameters.

## METHODS

This interventional study adhered to the tenets of the Declaration of Helsinki and was approved by the Institutional Review Board of the Federal University of São Paulo. In addition, all participants provided written informed consent. We included all patients who had an indication for trabeculectomy in the next few months. Trabeculectomy was performed by different surgeons in a standardized manner. The trabeculectomy technique was the same for all patients and consisted of topical or peribulbar anesthesia, fornix-based dissection of conjunctiva and tenon, application of mitomycin C in the subtenonian space for three minutes, confection of the scleral flap, trabeculectomy (with punch instrument), and application of flow-control sutures (Nylon 10-0) in the borders of the scleral flap, based on the surgeon’s intraoperative impression of flow control. All patients were treated with loteprednol etabonate ophthalmic suspension 0.5% four times a day for 1 week before trabeculectomy. Data from the baseline (day of surgery) and the follow-up visit (2 weeks after surgery) were included in the analysis.

### Study participants

A total of 31 patients with open-angle glaucoma were included in the study. Only patients with glaucoma who had an indication for trabeculectomy from March 2018 until December 2018 were included. Glaucoma was defined as the presence of repeatable (≥three consecutive) abnormal standard automatic perimetry (SAP) test results on the 24-2 program of the visual field (Humphrey Field Analyzer; Carl Zeiss Meditec, Inc) or progressive glaucomatous optic disk changes noted on masked examination of stereo photographs, regardless of the results of the SAP testing. Abnormal SAP was defined as the presence of a pattern standard deviation index outside the 95% confidence limits or glaucoma hemifield test results outside the reference range. The exclusion criteria were (1) systemic diseases affecting the ocular surface, (2) any acute disease affecting the ocular surface (e.g., acute conjunctivitis), (3) use of contact lenses, (4) previous ocular surgery or trauma, and (5) history of OSD prior to starting hypotensive agents or a history of chronic BAK exposure.

### Demographic and socioeconomic parameters

To avoid bias in our main results, we evaluated patients’ socioeconomic and clinical parameters. All par ticipants completed a questionnaire in which were asked to provide information on the following items: gender (female yes/no), ethnicity (black yes/no), marital status (married yes/no), and educational level (at least high school degree yes/no). These variables were added because they could affect the patient’s perception of QoL. To evaluate possible morbidities, the presence or history of several diseases was investigated, such as high blood pressure, diabetes mellitus, arthritis, heart disease, stroke, depression, cancer, and asthma. The comorbidity index was calculated from the sum of some scores given to each item. Patient visual acuity (VA) and number of topical medications were also collected from all patients. VA was measured using the Early Treatment Diabetic Retinopathy Study, and logMAR calculations were also included in the analysis.

### Ocular surface disease index

All patients answered two questionnaires: a general epidemiological questionnaire and a questionnaire called the Ocular Surface Disease Index (OSDI)^(14-16)^. The questionnaire was validated in Brazil by Prigol et al.^(17)^ and is a 12-item scale divided into three categories with the aim of assessing symptoms related to dry eye disease and their effect on vision^(18,19)^. The first part is associated visual function (questions 1 to 5), the second part with ocular symptoms (questions 6 to 9), and the third with environmental triggers (questions 10 to 12). Each item is scored on a scale ranging from 0 to 4 according to the frequency of the symptoms: 0 indicates symptoms none of the time; 1, some of the time; 2, half of the time; 3, most of the time; and 4, all the time. The total OSDI score is calculated on the basis of the following formula: OSDI = [(sum of the score for all the questions answered) × 100]/[(total number of questions answered) × 4]. The total score ranges from 0 to 100, with higher scores indicating worse OSD.

### Clinical evaluation

All patients underwent a detailed ophthalmic examination including best corrected VA, slit-lamp examination, IOP (Goldmann), and fundoscopy. To evaluate OSD, we used tear breakup time (BUT), bulbar redness (BR), and the presence/absence of keratitis. BUT was classified as (1) less than 5 seconds, (2) between 5 and 10 seconds, and (3) greater than 10 seconds. BR was scored from 0 to 4 according to the Institute for Eye Research-Brien Holden Vision Institute scales^(20,21)^ using comparative photos in which 0 indicated an absence of BR; 1, very slight BR; 2, slight BR; 3, moderate BR; and 4, severe BR. Keratitis was evaluated by staining the cornea cell surface with fluorescein eyedrops and classified accor ding to the absence or presence of kereatitis (slight, moderate, or severe). The ophthalmologic examination was performed last to avoid any influence of fluorescein on the stability of the tear film or the ocular surface.

### Keratograph analysis

The Keratograph 5M (Oculus, Wetzlar, Germany) is a noninvasive imaging device that uses infrared light and has automated features that do not require topical anesthesia, fluorescein staining, white light, or manual timing^(22)^. It was used to objectively analyze the ocular surface features by quantifying tear meniscus height (TMH), BR by keratograph (BR-K), noninvasive tear BUT (NIKBUT), and meibomian glands (meibography).

Tear meniscus height was analyzed using Oculus TMH tool images, which were graded perpendicular to the lid margin at the central point relative to the pupil center and measured in millimeters. NIKBUT was measured by using infrared light video from the NIKBUT tool, which measures time in seconds until the first breakup of tears (NIKBUT FIRST), as well as using a graph that shows the location of the first break. BR-K was assessed and scored automatically by the keratograph using a photo of the anterior biomicroscopy. The light scan detects vessels in the conjunctiva and evaluates the degree of redness. The keratography scale of BR was scored from 0 to 4 according to the Institute for Eye Research-Brien Holden Vision Institute scales^(22)^ using comparative photos of BR, with 0 indicating no BR; 1, very slight BR; 2, slight BR; 3, moderate BR; and 4, severe BR.

For the meibomian evaluation, we used a meibography tool to generate IR images of the tarsal conjunctiva. The upper and lower eyelid were everted, and manual grading of the meibomian gland images was performed using a meiboscale (degrees from 0 to 4): 0 eyes with total meibomian integrity, 1 to an area of meibomian loss less than 25%, 2 to an area of loss from 25% to 50%, 3 to an area of loss from 51% to 75%, and 4 to an area of loss more than 75% of the total area.

### Statistical analysis

The descriptive analysis included the mean and standard deviation for variables with a normal distribution, whereas variables that were not distributed normally were presented as the median. We used the skewness-kurtosis test to confirm normality. The t test was used for multiple comparisons between pre-and postoperative measurements, and for non-normal variables, the corresponding nonparametric test (Wilcoxon rank test) was performed. Percentages were used to describe categorical values and achieve better comparators between the two groups. All statistical analyses were performed using the available software Stata version 13 (StataCorp LP, College Station, TX). The alpha level (type I error) was set at 0.05.

## RESULTS

We included 31 eyes of 31 patients with glaucoma. The mean age of the glaucoma patients was 69.90 ± 10.77 years. Of the sample, 18 (58.06%) were female pa tients and 10 (32.26%) were of Caucasian ancestry. The average VA was 0.40 ± 0.34 logMAR. The average comorbidity index and number of oral medications were 1.10 ± 0.91 and 1.32 ± 0.48, respectively. It is important to point that, for the first month after the surgery, only topical antibiotic eyedrops (monofloxacin 0.5%) four times a day and steroid eyedrops (prednisolone acetate 1%) were prescribed for all subjects. Table 1 summarizes the demographic and clinical findings of our study.

**Table 1:** Demographic and Clinical Findings in the Glaucoma Subjects

Glaucoma Subjects (N=31)	
Age ± SD (years)	69.90 ± 10.77
Gender (%)	
Female	18 (58.06%)
Male	13 (41.94%)
Race (%)	
Caucasian	10(32.26%)
Black	2 (6.50%)
Other	19(61.29%)
Visual acuity ± SD (logMar)	0.40 ± 0.34
Number of oral medications ± SD	1.32 ± 0.48
Number of topical medications ± SD	2.90 ± 0.75
Comorbidities index ± SD	1.10 ± 0.91
Marital status (married, yes %)	10(32.26%)
Level of education (%)	
>High school	26 (83.87%)
<High school	5 (16.13%)

SD= standard deviation; dB= decibels; %= percentage; N= number.

For the subjective analysis, the overall OSDI prevalence rate was 27.20 ± 17.56 units. The mean value for the questions associated with visual function (questions 1 to 5) was 7.42 ± 5.37 units. The mean value for the questions associated with ocular symptoms (questions 6 to 9) was 2.68 ± 2.41 units, and the mean value for the questions related to environmental triggers (questions 10 to 12) was 2.97 ± 3.87 units. Table 2 summarizes the overall results from the OSDI.

**Table 2:** Ocular surface disease index and subscale results

Parameters, units (Mean ± SD)	Glaucoma subjects (*N*=31)
Overall ocular surface disease index	12.39 ± 7.16
General health subscale	7.55 ± 4.33
General vision subscale	3.81 ± 4.06
Ocular pain subscale	1.10 ± 1.33

SD= standard deviation; *N*= number.

The clinical evaluations showed that 58.00% of patients had an absence of BR before surgery and 48.39% of patients had slight BR after surgery (*p*=0.056). Of the patients, 42.00% patients had slight keratitis before surgery, and 39.00% also had slight keratitis after the surgery (p=0.787). A total of 51.60% of patients had BUT between 5 and 10 seconds before surgery, and 48.39% of patients had BUT between 5 and 10 seconds after the surgery (p=0.537).

**Figure 1:**
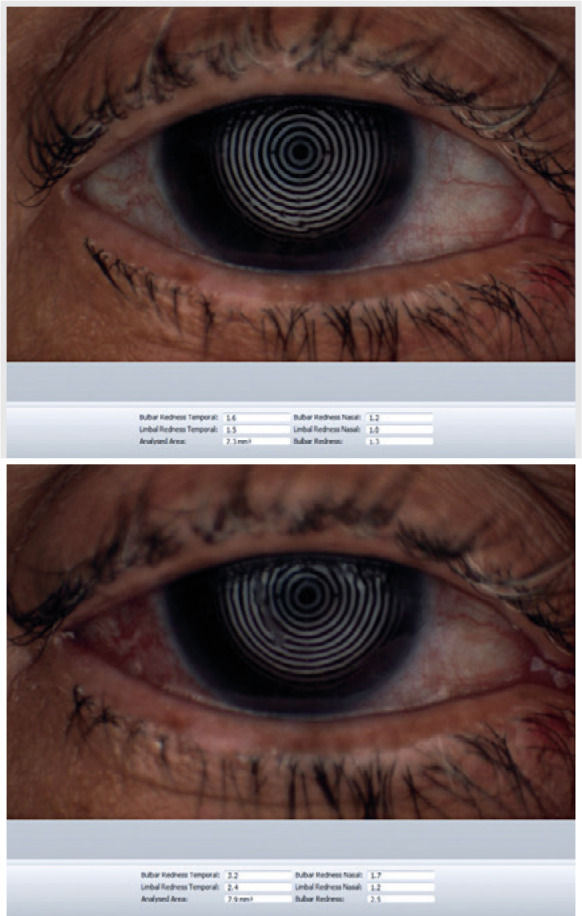
Keratograph of a 68-year-old male patient showing bulbar redness, preoperative and postoperative, respectively.

**Figure 2:**
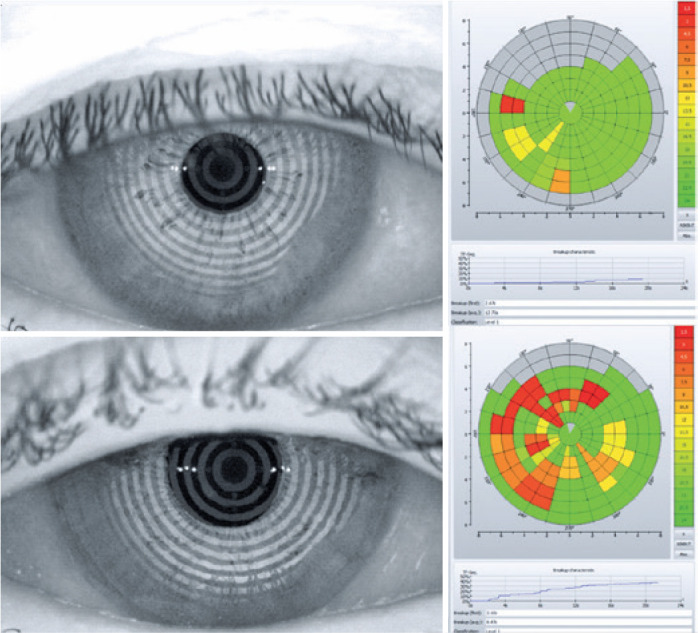
Keratograph of a 68-year-old male patient showing bulbar redness, preoperative and postoperative, respectively.

**Figure 3:**
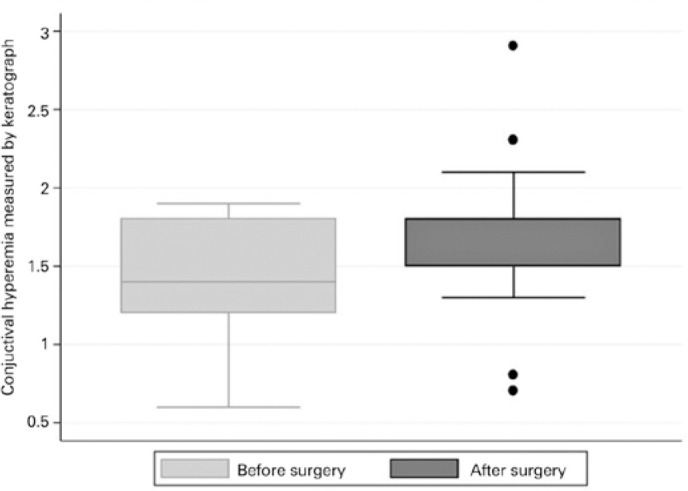
Boxplot depicting the distribution of conjunctival hyperemia measured by keratograph. Box: median and interquartile range (IQR). The whiskers show the maximum and minimum 1.5 IQR.

**Figure 4:**
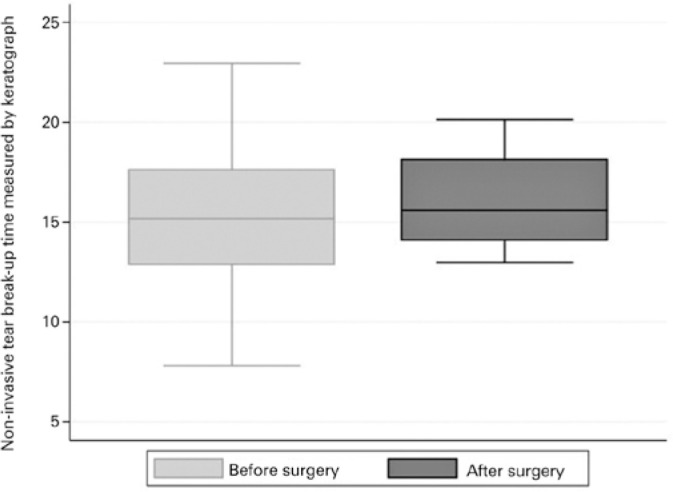
Boxplot depicting the distribution of noninvasive tear breakup time measured by keratograph. Box: median and interquartile range (IQR). The whiskers show the maximum and minimum 1.5 IQR.

For the keratograph analysis (TMH, BR-K, NIKBUT, meibography quantification for the upper and lower eyelid), the only two parameters that were significantly different before and after trabeculectomy was the BR-K (Figure 1) and the average of the NIKBUT (Figure 2). After trabeculectomy, patients presented with more conjunctival hyperemia compared with before surgery (1.42 ± 0.36 and 1.68 ± 0.48, respectively, p=0.013; Figure 3). After trabeculectomy, patients presented shorter NIKBUT compared with before the surgery (16.22 ± 2.37 and 14.98 ± 3.1, respectively, p=0.041; Figure 4).

There was no significant difference in TMH or quantification of meibography before and after treatment (p>0.05 for all comparisons).

## DISCUSSION

In this study, we found that although the use of loteprednol etabonate ophthalmic suspension 0.5% may improve OSD in glaucoma patients, our patients presented with more conjunctival hyperemia as measured by keratograph analysis. Previous studies have already addressed this issue in glaucoma patients and reported an improvement in OSD after administration of loteprednol etabonate. However, because these patients had recently undergone trabeculectomy surgery, they might present with worse OSD. To our knowledge, this preliminary study is the first to attempt to compare the same patient before and after trabeculectomy using keratograph analysis.

The presence of conjunctival hyperemia measured by clinical evaluation has been discussed extensively in the past years. For example, the Glaucoma Adherence and Persistency Study (GAPS)^(23)^ evaluated 300 open-angle glaucoma patients without previous treatment (surgical or clinical) in the past 6 months using a data interview. The study showed that hyperemia was the most common side effect of topical eyedrops, and it was responsible for the stopping or switching of medication in 63% of patients, especially in those using prostaglandin. Park et al.^(24)^ examined the common side effects of topical an tiglaucomatous medications as well as the factors affecting compliance with glaucoma treatment and found that conjunctival injection, stinging sensation, and blurred vision were the most frequent uncomfortable side effects. Using keratograph technology, Pérez Bartolomé et al.^(25)^ compared the ocular redness from 211 eyes of 211 patients with open-angle glaucoma or ocular hypertension using topical medication with 51 eyes of healthy volunteers and found statistically significant results. In addition, Pérez et al.^(25)^ showed that higher redness scores were recorded in the medication group (p<0.01 for all scores).

The present study found no difference in terms of keratitis before and after surgery using clinical assessment or keratograph analysis. After analyzing almost 400 eyes, Ono et al.^(26)^ found that the severity of OSD after trabeculectomy is related to its intensity before the surgery and also reported no statistically significance difference in keratitis or tear BUT before and after the procedure. Zhong et al.^(27)^ recently analyzed OSD after trabeculectomy using keratograph technology. Their study recruited 81 patients without previous OSD with an indication of trabeculectomy and followed them through 3 months postoperatively. Results showed worse tear BUT and fluo rescein stain in the first month, with partial recovery at the third month. However, this final recovery was still worse than the baseline. Many studies have proven that it is not just glaucoma eye drops that contribute to OSD, but also that the intraoperatively use of mitomycin C is a factor^(27,28)^. However, this is the first study that has attempted not only to analyze the effect of preoperative steroid eye drops on OSD but also to objectively quantify these findings using keratograph technology.

It is important to address some specific points of this study. Despite being the first study to use keratographs to investigate OSD in glaucoma patients, this was a cross-sectional study that enrolled a small sample size. In addition, although the clinical examinations were performed by only one ophthalmologist and findings were classified based on a well-established scale, issues remain regarding the study’s subjectiveness. Another point that must be considered is the fact that patients might present with worse OSD because of recent trabeculectomy surgery, as any surgery can be considered a “trauma” for the eye and stimulate proinflammatory agents.

In conclusion, although loteprednol etabonate ophthalmic suspension 0.5% has been associated with an improvement in OSD in glaucoma patients, our sample presented with more conjunctival hyperemia as measured by keratograph analysis. Because these patients recent underwent trabeculectomy surgery, they might present with worse OSD.
